# Photodynamic Inactivation of Mammalian Viruses and Bacteriophages

**DOI:** 10.3390/v4071034

**Published:** 2012-06-26

**Authors:** Liliana Costa, Maria Amparo F. Faustino, Maria Graça P. M. S. Neves, Ângela Cunha, Adelaide Almeida

**Affiliations:** 1 Department of Biology and CESAM, University of Aveiro, 3810-193 Aveiro, Portugal; Email: lcosta@ua.pt (L.C.); acunha@ua.pt (A.C.); 2 Department of Chemistry and QOPNA, University of Aveiro, 3810-193 Aveiro, Portugal; Email: faustino@ua.pt (M.A.F.F.); gneves@ua.pt (M.G.P.M.S.N.)

**Keywords:** bacteriophages, mammalian viruses, photodynamic therapy, photosensitizer, viral photoinactivation process

## Abstract

Photodynamic inactivation (PDI) has been used to inactivate microorganisms through the use of photosensitizers. The inactivation of mammalian viruses and bacteriophages by photosensitization has been applied with success since the first decades of the last century. Due to the fact that mammalian viruses are known to pose a threat to public health and that bacteriophages are frequently used as models of mammalian viruses, it is important to know and understand the mechanisms and photodynamic procedures involved in their photoinactivation. The aim of this review is to (i) summarize the main approaches developed until now for the photodynamic inactivation of bacteriophages and mammalian viruses and, (ii) discuss and compare the present state of the art of mammalian viruses PDI with phage photoinactivation, with special focus on the most relevant mechanisms, molecular targets and factors affecting the viral inactivation process.

## Nomenclature

AlPcS_4_: Aluminum phthalocyanine tetrasulfonate
AZT: Azidothymidine
BVDV: Bovine viral diarrhea virus
DMTU: Dimethylthiourea
EMCV: Encephalomyocarditis virus
HAV: Hepatitis A virus
HBV: Hepatitis B virus
HCV: Hepatitis C virus
HIV: Human immunodeficiency virus
HPV: Human papillomatosis virus
HSV: Herpes simplex virus
LED: Light emitting diode
MB: Methylene blue
NM: Not mentioned
NQ: Not quantified
Pc_4_: Silicon phthalocyanine
PDI: Photodynamic inactivation
PS: Photosensitizer
ROS: Reactive oxygen species
SFV: Semliki Forest virus
SHV: Suid herpes virus
SOD: Superoxide dismutase
SSB: Singlet strand breaks
Tri-Py^+^-Me-PF: 5-(pentafluorophenyl)-10,15,20-tris(1-methylpyridinium-4-yl)porphyrin tri-iodide
VSV: Vesicular stomatitis virus
VZV: Varicella zoster virus
^1^O_2_: Singlet oxygen
^3^O_2_: Molecular oxygen
^1^PS: Ground state photosensitizer
^3^PS^*^: Triplet excited state photosensitizer

## 1. Introduction

Humans are exposed to pathogenic viruses through various routes and the development of viral-induced diseases is a common occurrence.

Although the transmission of viral diseases has been reduced by the development of good water supplies and hygienic-based procedures for a whole range of human activities [1], pathogenic viruses are still the causative agents of many diseases in humans and other species. The most usual human diseases caused by viruses include the common cold (coronaviruses), influenza (influenza viruses), chickenpox (varicella zoster virus), cold sores (herpes simplex virus), gastroenteritis and diarrhoea (caliciviruses, rotaviruses and adenoviruses) [2,3]. Pathogenic viruses are also implicated in serious diseases, such as Ebola (Ebola virus), AIDS (immunodeficiency viruses), avian influenza and sudden acute respiratory syndrome (SARS) (SARS-coronavirus), and they are also an established cause of cancer (papillomavirus, hepatitis B and C viruses, Epstein–Barr virus, Kaposi’s sarcoma-associated herpes virus, human T-lymphotropic virus, and Merkel cell polyomavirus) [4]. 

The enhanced implication of viruses in severe infectious diseases and the increasing knowledge about the complex mechanisms of viral pathogenesis have greatly contributed to the rapid development of antiviral drugs. Consequently, the use of antivirals has largely increased in the last years and resistance to antiviral drugs is now well documented for several pathogenic viruses [5,6,7,8,9,10]. Moreover, as viruses are genetically flexible, they may mutate quickly and mutations come as no surprises, leading to the development of resistance to conventional antiviral drugs. Consequently, the emergence of antiviral drug can become a great problem, such the resistance observed for bacteria relative to antibiotics. So, alternative methods unlikely to cause resistance are required. Photodynamic inactivation (PDI) of viruses represents a promising and inexpensive potential alternative to meet that need.

The sensitivity of viruses to photodynamic procedures was reported in the 1930s [11,12] but only within the last 30 years, with the development of new active molecules, namely photosensitizers (PS), and an increment of light technologies (lasers, LED, portability, *etc.*), have photodynamic techniques for the inactivation of viruses received growing attention [13]. Most of the clinical applications of PDI for treatment of infections have so far been directed to viral lesions [14]. Clinical PDI was first applied to the treatment of herpes infection in the early 1970s [15], particularly for herpes genitalis. Since then, a great variety of viruses has been effectively inactivated by photodynamic treatment using *in vitro* conditions [16] but, considering the clinical use of viral PDI, the procedures are limited to the treatment of papillomatosis, caused by human papillomatosis virus (HPV), like laryngeal papillomatosis [17] and epidermodysplasia verruciformis [18] and, in a small scale, to the treatment of viral complications in AIDS patients [19,20]. However, considerable progress has been made in the viral photodynamic disinfection of blood products. The major threat of viral contamination in blood and blood products comes from the immunodeficiency viruses (HIV) [21], hepatitis viruses [21,22,23], cytomegalovirus [23], human parvovirus B19 [24] and human T-cell lymphotropic virus type I and type II [23]. HIV has been inactivated *in vitro* following a photodynamic procedure [25,26,27,28,29,30,31,32,33,34,35,36,37,38,39]. The photoinactivation of hepatitis viruses in blood products has also been successfully tested against the hepatitis C virus (HCV) [37,40,41,42], hepatitis B virus (HBV) [43] and hepatitis A virus (HAV) [44]. Inactivation of cytomegalovirus [45], human parvovirus B19 [46] and human T-cell lymphotropic virus [47] in blood products was also efficiently achieved after photodynamic treatment.

The availability of a simple and quantitative assay to follow the viral photoinactivation process is important. Traditional viral quantification techniques, such as *in vitro* viral cultures, are time-consuming and labor-intensive processes. Molecular quantitative methods such as nucleic acid amplification procedures, including real time PCR, are rapid and sensitive but detect only viral nucleic acid and do not determine infectivity. When the virucidal properties of different photosensitizing compounds are initially evaluated, bacteriophages can be useful as surrogates of mammalian viruses. The reasons for their use are: (i) the detection methods are much simpler, faster and cheaper than those of mammalian viruses, avoiding the advanced facilities and equipment needed for propagating human pathogens; (ii) they are non-pathogenic to humans; (iii) they can be grown to higher titers than most mammalian viruses and, therefore, enhancing the sensitivity of the assay; (iv) the results of bacteriophages assays are available within several hours post-inoculation, instead of the days or weeks required by mammalian viruses infectivity-based assays; (v) they are at least as resistant as the mammalian viruses to environmental factors and to water treatment [48].

It has been shown that enveloped viruses are significantly more sensitive to photodynamic destruction than non-enveloped viruses [49,50]. As most of the bacteriophages are non-enveloped, they are more difficult to suffer photoinactivation than the enveloped viruses. In general, this property makes them good indicators to evaluate the efficiency of viral PDI. A PDI protocol that is effective to inactivate a non-enveloped phage will most likely be effective against enveloped mammalian viruses.

Several bacteriophages were used in photoinactivation studies as surrogates for mammalian viruses, e.g., MS2 [44], M13 [51,52], PM2 [53], Qβ [54,55,56], PRD1 [57], λ [58,59], φ6 [60], R17 [60], *Serratia* phage *kappa* [61], T5 [62], T3 [63], T7 [57,64] and T4-like [65,66,67,68], and the results show that they are effectively photoinactivated.

## 2. Antimicrobial PDI

PDI is a simple and controllable method for the inactivation of microorganisms based on the production of reactive oxygen species (ROS) (free radicals and singlet oxygen). This technology requires the combined action of oxygen, light and a photosensitizer (PS), which absorbs and uses the energy from light to produce those ROS [69]. Therefore, the photodynamic effects depend on multiple variables including: the structural features of the PS, the concentrations of PS and molecular oxygen, and the properties of the light used (e.g., wavelength, type, dose and fluence rate) [66,67,69,70,71,72]. Changes in any of these parameters will affect the rate of microbial photoinactivation [66,67,73,74].

**Figure 1:**
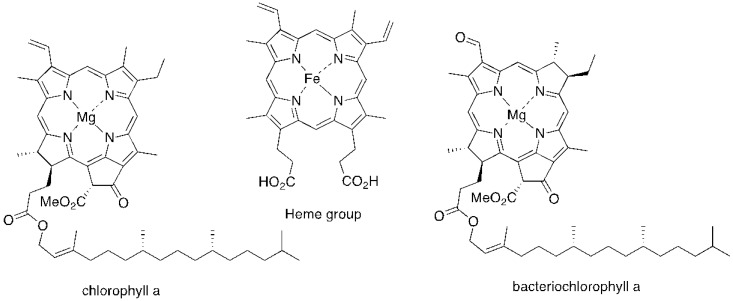
Structure of some tetrapyrrolic macrocycles with natural occurrence.

**Figure 2:**
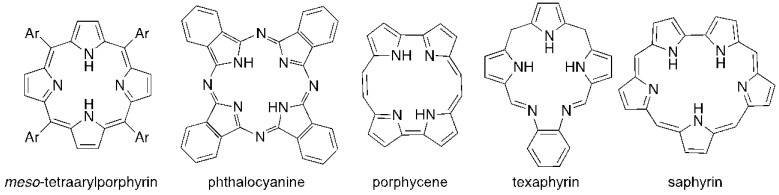
Skeletons of some synthetic pyrrolic macrocycles used as photosensitizers.

**Figure 3:**
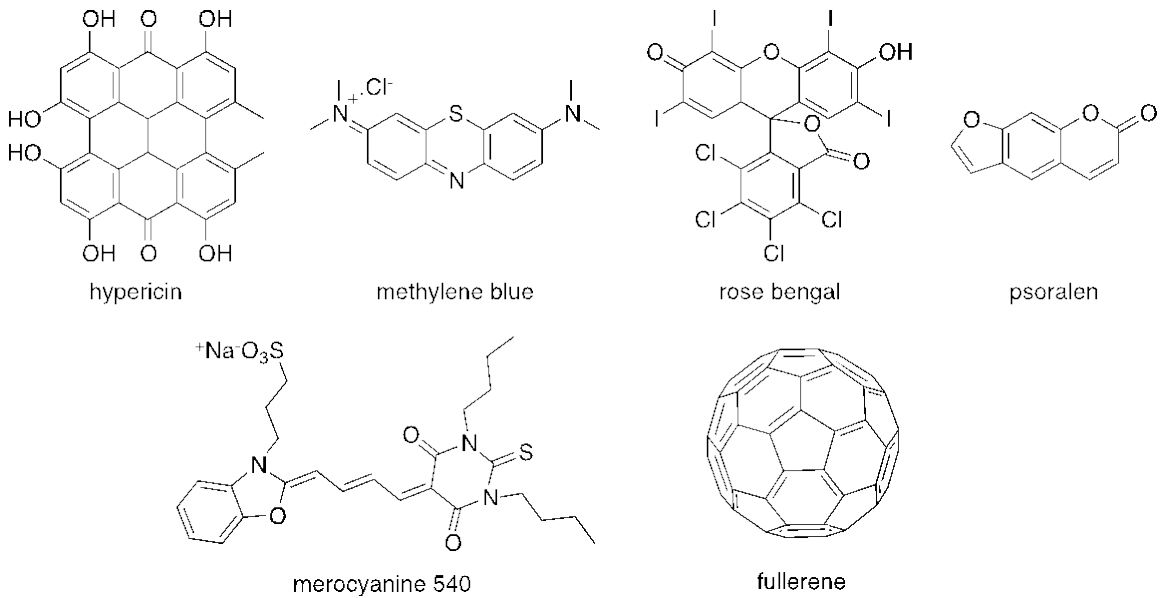
Structure of some non-tetrapyrrolic photosensitizers.

The majority of PS used in PDI is derived from tetrapyrrolic macrocycles known as porphyrins. These chromophores and their analogs, such as chlorins and bacteriochlorins, are involved in very important biological functions, such as respiration (heme group) and photosynthesis (chlorophyll and bacteriochlorophyll (Figure 1). Based on these macrocycles, the scientific community was able to develop a number of synthetic analogs, such as *meso*-tetraarylporphyrins, phthalocyanines, texaphyrins, porphycenes and saphyrins, which proved to have very promising features for being used as PS (Figure 2) [16]. Also, non-tetrapyrrolic derivatives, such as the naturally occurring hypericin, or synthetic dyes like toluidine blue O, rose bengal, eosin, methylene blue (MB) and fullerenes, were considered in many PDI studies (Figure 3) [71].

In order to be efficient, photosensitizing agents used for viral PDI must bind specifically to vital viral components, such as lipid envelope (when present), the protein coat or to the nucleic acids [55].

The efficiency of mammalian viruses and bacteriophages PDI has been described for porphyrin derivatives, chlorin derivatives, chlorophyll derivatives, phthalocyanine derivatives, hypericin, methylene blue, rose bengal, merocyanine 540, proflavine, and fullerene derivatives (Table 1).

**Table 1:** Some PS used for mammalian viruses and bacteriophages PDI.

Photosensitizer	Microorganism	PDI	Reference
**Mammalian viruses**			
Hematoporphyrin derivative	HSV-1	7 log	[75]
	HSV-1	<0.8 log	[36]
Uroporphyrin	Adenovirus	7 log	[76]
Natural metalloporphyrin derivatives	HIV-1	<0.8 log	[36]
Chlorophyll derivatives	VSV	~6 log	[77]
7-despropionate-7-hydroxypropylmesopyropheophorbide a	BVDV	~5 log	[78]
	EMCV	~0.2 log	
Benzoporphyrin derivative monoacid ring A	HIV-1	>4 log	[33]
Glycoconjugated *meso*-tetraarylporphyrin derivatives	HSV-1	6 log	[79]
	HSV-2	6 log	
Metallo tetrasulfonated *meso*-tetraarylporphyrin derivatives	HIV-1	≤2 log	[36]
Tetrasulfonated *meso*-tetraarylporphyrin derivatives	HIV-1	≤2 log	[36]
	HAV	~4 log	[44]
*meso*-Tetrakis(1-methylpyridinium-4-yl)porphyrin	HAV	~4 log	[44]
*meso*-Tetrakis(1-butylpyridinium-4-yl)porphyrin	HAV	>3.8 log	[44]
*meso*-Tetrakis(1-octylpyridinium-4-yl)porphyrin	HAV	>3.9 log	[44]
Cationic β-vinyl substituted *meso*-tetraphenylporphyrin derivatives	HSV-1	<3 log	[80]
Aluminum dibenzodisulfophthalocyanine	HIV-1	3.7 log	[49]
Aluminum phthalocyanine tetrasulfonate	HIV-1	>5 log	[49]
	VSV	4.2 log	[82]
	Adenovirus	4 log	[76]
Silicon phthalocyanine derivative	VSV	4 log	[82]
Cationic phthalocyanines	HIV-1	>5 log	[49]
	HSV-1	≥5 log	[83]
Hypericin	HIV-1	NQ	[30]
	VSV	4-5 log	
	Influenza virus	NQ	
	Sendai virus	NQ	
Methylene blue	VSV	4.7 log	[81]
	HSV-1	5 log	[84]
	SHV-1	2.5 log	[84]
	HCV	<2 log	[41]
	HIV-1	<2 log	[41]
	Adenovirus	7 log	[76]
	Dengue virus	5–6.4 log	[74]
	Enterovirus 71	~8 log	[85]
	Vaccinia virus	5 log	[86]
Phenothiazine derivatives	VSV	>4.4 log	[60]
Rose bengal	Vaccinia virus	5 log	[86]
	HIV-1	NQ	[30]
	VSV	4–5 log	
	Influenza virus	NQ	
	Sendai virus	NQ	
	Adenovirus	7 log	[76]
Buckminsterfullerene	SFV	7 log	[50]
	VSV	7 log	
Merocyanine 540	HSV-1	5–6 log	[45]
**Bacteriophages**			
Glycoconjugated *meso*-tetraarylporphyrins	T7 phage	<3 log	[64]
	T7 phage	<3.5 log	[87]
Tetrasulfonated *meso*-tetraarylporphyrin derivatives	MS2 phage	>3.8 log	[44]
*meso*-Tetrakis(1-methylpyridinium-4-yl)porphyrin	λ phage	<7 log	[58]
	MS2 phage	>4.1 log	[44]
	T4 phage	7 log	[66,67]
	T7 phage	<4 log	[88]
5-(pentafluorophenyl)-10,15,20-tris(1-methylpyridinium-4-yl)porphyrin	T4 phage	7 log	[66,67,68]
5-(4-methoxicarbonylphenyl)-10,15,20-tris(1-methylpyridinium-4-yl)porphyrin	T4 phage	7 log	[66]
5-(4-carboxyphenyl)-10,15,20-tris(1-methylpyridinium-4-yl)porphyrin	T4 phage	3.9 log	[66]
5,10-bis(4-carboxyphenyl)-15,20-bis(1-methylpyridinium-4-yl)porphyrin	T4 phage	1.4 log	[66]
5,15-bis(4-carboxyphenyl)-10,20-bis(1-methylpyridinium-4-yl)porphyrin	T4 phage	1.2 log	[66]
5,10,15-tris(1-methylpyridinium-4-yl)-20-phenylporphyrin	T7 phage	1.7 log	[88]
Methylene blue	*Serratia* phage *kappa*	>4 log	[61]
	M13 phage	2.2 log	[52,81]
	f2 phage	5 log	[56]
	Qβ phage	7–8 log	[56]
	Qβ phage	7–8 log	[89]
Phenothiazine derivatives	R17 phage	4–7 log	[60]
	φ6	4–6.5 log	
Rose bengal	PRD1 phage	~3.5 log*	[57]
	T7 phage	~4.5 log*	
Riboflavin	λ phage	<4 log	[59]
Proflavine	*Serratia* phage *kappa*	4 log	[61]
	T3 phage	7–11 log	[63]
Polyhydroxylated fullerene	MS2 phage	~4 log	[90]
	PRD1 phage	~2.5 log*	[57]
	T7 phage	~3.5 log*	
	MS2 phage	~5 log*	

*log(N/N0)

Besides this, viral PDI has also been described for phthalocyanine derivatives [81], methylene blue [53,62,91,92], toluidine blue O [53,62,93], neutral red [93], proflavine [93], azure B [53] and merocyanine 540 [45,47,94].

## 3. Mechanisms of Photodynamic Inactivation

**Figure 4:**
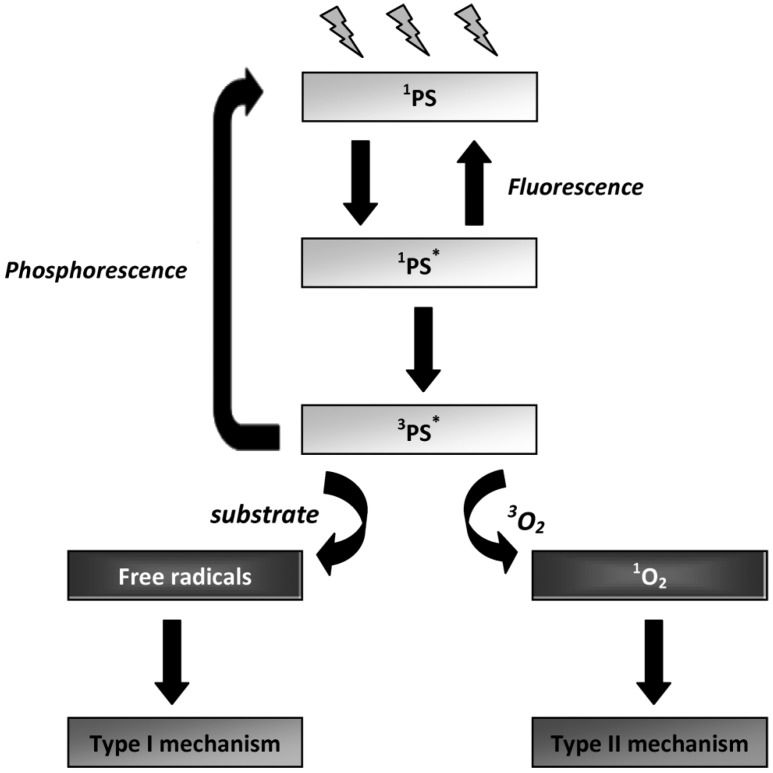
Schematic representation of the photosensitization process (adapted from [97]).

The mechanisms of PDI are based on the ability of the PS to absorb energy from light and transfer that energy to molecular oxygen. In the dark, the electronic configuration of a PS exists in the so-called ground state. The absorption, by the PS, of a photon at an appropriate wavelength initially leads to the production of an unstable, electronically-excited state of the PS molecule (the lifetime of this state ranges from 10^−9^ to 10^−6^ s) [95]. The excited PS molecule can then decay to the ground state by emission of light (radiative pathway - fluorescence) or by intersystem crossing, affording the excited triplet state which has a longer lifetime (10^−3^ to 10 s) [95]. At this point, the PS can reach the ground state either by spin inversion followed by phosphorescence emission, or by a non-radiative process. Due to the longer lifetime of the PS triplet state, this excited state can also react in one of two ways (Figure 2): by initiating photochemical reactions that can directly generate reactive oxygen species (ROS) (type I pathway), or indirectly by energy transfer to molecular oxygen (type II pathway), leading to the formation of singlet oxygen (Figure 4). These events afford toxic species which are responsible for the irreparable oxidative damages induced to important biological targets [1,69,95,96].

### 3.1. Type I and Type II Mechanisms

Type I mechanism involves hydrogen-atom abstraction or electron-transfer between the excited PS and a substrate, yielding free radicals [Equations (1) and (2)]. These radicals can react with oxygen to form active oxygen species, such as the superoxide radical anion [Equation (3)]. Superoxide is not particularly reactive in biological systems but, when protonated, can lead to the production of hydrogen peroxide and oxygen [Equations (4) and (5)] or highly reactive hydroxyl radicals [Equations (6)–(8)] [98]. Type II photooxidation is considerably less complex mechanistically than type I and in general there are far fewer products [99]. In this pathway, the excited triplet state PS (^3^PS*) can transfer the excess energy to molecular oxygen (^3^O_2_) and relax to its ground state (^1^PS) creating an excited singlet molecular oxygen (^1^O_2_) [Equation (9)] [69]. ^1^O_2 _is highly electrophilic and can interact with numerous enzymes, leading to the inhibition of protein synthesis and molecular alteration of DNA strands, which alters the transcription of the genetic material during its replication (mutagenic effect) and, in this way, leading to microbial death [Equation (10)] [98,100]. Like nucleic acids and proteins, unsaturated lipids are also prominent targets of ^1^O_2 _and free radical attack. Lipid peroxidation-ensuing reactions can alter surrounding proteins, nucleic acids and other molecules, in addition to the lipids themselves [98]. Therefore, it is likely that damage of different kinds caused to the viral envelope is important in the process of microbial inactivation [13]. 


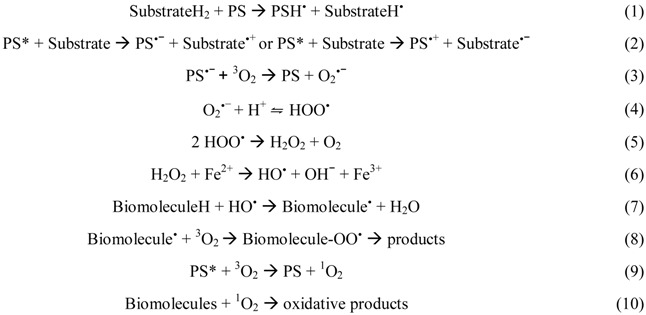


Both type I and type II mechanisms can occur simultaneously or exclusively, and the ratio between these processes depends on the PS used and on the concentrations of substrate and oxygen [95]. The competition between organic substrates and molecular oxygen for the ^3^PS^*^ determines whether the reaction pathway is type I or type II and the predominant mechanism can be changed during the course of the PDI process [101]. 

### 3.2. Evaluation of the Specific Involvement of Type I and Type II Mechanisms

An important goal in the investigation of viral PDI is to identify the type of mechanism involved (type I or type II) in the presence of a selected PS [102]. The simple detection of a reactive species does not necessarily explain the mechanism by which a specific PS induces the toxic effect. It is generally easier to draw a negative conclusion, *i.e.*, if singlet oxygen is absent, it cannot be the reactive species responsible for the photodynamic effect [103]. The simplest approach for determining whether singlet oxygen (type II mechanism) or free radicals (type I mechanism) is involved in the photodynamic process is to study the inhibitory effects of various scavengers, *i.e.*, compounds that can intercept these ROS at high rates and in a putatively selective manner [99,101,104].

#### 3.2.1. Type I Mechanism Scavengers

A first line of defence against ROS is, of course, the protection against their formation. However, the interception of the damaging species once formed, to prevent it from further deleterious reactions, is also a deactivation strategy of defence. In general, free radical scavengers neutralize the radical species by donating one of their own electrons. The quenching agents themselves are not particularly toxic before and after the electron donation [105].

Three different types of quenching are possible, which include the transfer of the radical character with the formation of a reactive scavenger-derived radical; trapping of free radicals with the formation of a stable or inert free radical trap; and molecules which mimic quenching enzyme activities. In general, scavenger molecules either prevent free radicals from being formed or remove them before they can damage vital molecular components [105].

Several free radical scavengers have been used to evaluate the specific involvement of type I mechanism during mammalian viruses and bacteriophages PDI with different PS (Table 2).

**Table 2:** Free radical scavengers used in mammalian viruses and bacteriophages PDI.

PS	Scavenger	Microorganism	Scavenger protection	Reference
**Mammalian viruses**				
Aluminum phthalocyanine tetrasulfonate	Reduced glutathione	VSV	Little/no effect	[106]
	Mannitol		Little/no effect	
	Glycerol		Little/no effect	
	SOD		Little/no effect	
Polyhydroxylated fullerene	Glutathione (2.0 mM)	SFV	no effect	[50]
		VSV	no effect	
	Hydroquinone (2.0 mM)	SFV	no effect	[50]
		VSV	no effect	
Merocyanine 540	Glutathione (10 and 30 mmol L^−1^)	HSV-1	30-50%	[45]
	Cysteamine (10 and 30 mmol L^−1^)		60-70%	
	SOD (1.5 to 29 U mL^−1^)		no effect	
Methylene blue	Mannitol (100 mM)	HSV-1	24%	[84]
	Glycerol (10 mM)		24%	
	SOD (300 U mL^−1^)		24%	
	Catalase (30 U mL^−1^)		24%	
**Bacteriophages**				
5,10,15-(4-β- D-glucosylphenyl)-20-phenylporphyrin	DMTU (0.1–5.0 mM)	T7 phage	44%	[64]
5,10.15,20-Tetrakis(4-β- D-glucosylphenyl) porphyrin	DMTU (0.1–5.0 mM)	T7 phage	79%	[64]
5,10,15-(4-β- D-galactosylphenyl)-20-(pentafluorophenyl)-porphyrin	DMTU (0.1–5.0 mM)	T7 phage	89%	[87]
5-(pentafluorophenyl)-10,15,20-tris(1-methylpyridinium-4- yl)porphyrin	D-mannitol (100 mM)	T4 phage	20%	[107]
		Qβ	no effect	
	L-cysteine (100 mM)	T4 phage	9%	[107]
5,10,15,20-tetrakis(1-methylpyridinium-4-yl)porphyrin	D-mannitol (100 mM)	T4 phage	no effect	[107]
Proflavine	L-cysteine (0.025 M)	T3 phage	75–80%	[63]
Polyhydroxylated fullerene	SOD	MS2 phage	no effect	[90]

##### 3.2.1.1. Free Radicals in PDI of Mammalian Viruses

Free radical species had, in general, little or no effect on the photoinactivation of the studied mammalian viruses (Table 2). In fact, it can be observed that the rate of inactivation of HSV [45,84,106], influenza virus [108], Semliki Forest virus (SFV) and VSV [50] in the presence of different PS and scavengers like glutathione, D-mannitol, glycerol, superoxide dismutase (SOD), catalase and hydroquinone was not significantly affected. Although this data suggest that free radicals are not major players in the viral inactivation process, the participation of type I reaction pathways cannot be ruled out, as was shown by the considerable level of protection afforded by glutathione and cysteamine when merocyanine 540 was used as PS for inactivation of HSV-1 [45].

##### 3.2.1.2. Free Radicals in PDI of Bacteriophages

The photoinactivation rate of some bacteriophages can be reduced in the presence of free radical scavengers, suggesting a contribution of radical species in the inactivation process (Table 2). In particular, it was reported that the inhibition of T7 phage photoinactivation in the presence of glycoconjugated *meso*-tetraarylporphyrins varied according to the structure of the PS and the concentration of dimethylthiourea (DMTU) [64,87]. In fact, T7 phage PDI by *meso*-tetrakis(4-β-D-glucosylphenyl)porphyrin [64] and 5,10,15-(4-β-D-galactosylphenyl)-20-(pentafluorophenyl)porphyrin [87] seemed to be mainly mediated by free radical species, as revealed by the protection effect of free radical scavenger DMTU, contrary to T7 phage photosensitization by 5,10,15-(4-β-D-glucosylphenyl)-20-phenylporphyrin, which revealed a significantly smaller contribution from type I mechanism. The highest inhibition was reached at about 1.0 mM of DMTU; further increase in scavenger concentration did not decrease the slope of photoinduced inactivation of phages. However, in spite of inhibiting the efficacy of the PS, DMTU did not completely inhibit T7 phage PDI [64,87]. Similar results were reported for T3 phage in the presence of L-cysteine as the scavenger and proflavine as the PS. However, the photoinactivation rate of MS2 by a polydroxylated fullerene was not affected by the presence of SOD, suggesting a negligible contribution of radical species, such as the superoxide radical anion [90]. T4-like phage PDI was also little or not affected by the presence of free radical scavengers L-cysteine and D-mannitol in the presence of porphyrin derivatives, leading to the conclusion that free radical species are not major participants in phage PDI [107].

#### 3.2.2. Type II Mechanism Quenchers

In general, the action of chemical singlet oxygen quenchers involves the reaction of singlet oxygen with the quenching agent, producing an oxidized product. Another possibility is the deactivation of singlet oxygen to ground state (^3^O_2_) by physical quenching, achieved by either energy or charge transfer, without consumption of oxygen or product formation [101,109]. Residues of histidine, tryptophan and tyrosine in proteins are considered to be major natural quenchers of singlet oxygen [110].

Several singlet oxygen quenchers have been used to evaluate the specific involvement of type II mechanism during viral PDI with different PS (Table 3).

**Table 3:** Singlet oxygen quenchers used on mammalian viruses and bacteriophage PDI.

PS	Quencher	Microorganism	Quencher protection	Reference
**Mammalian viruses**				
Aluminum phthalocyanine tetrasulfonate	Sodium azide	VSV	significant effect	[106]
	Tryptophan	VSV	Significant effect	
Rose bengal	β-carotene	Influenza virus	Significant effect	[108]
	Sodium azide			
Hypericin	Sodium azide	HIV	Significant effect	[111]
Methylene blue	Imidazole (5.0 and 10 mM)	HSV-1	55–75%	[84]
**Bacteriophages**				
5,10,15-(4-β- D-galactosylphenyl)-20-(pentafluorophenyl)porphyrin	Sodium azide (0.1–5.0 mM)	T7 phage	38%	[87]
5-(pentafluorophenyl)-10,15,20-tris(1-methylpyridinium-4-yl)porphyrin	Sodium azide (100 mM)	T4 phage	80%	[107]
		Qβ	39%	
	L-histidine (50 mM)	T4 phage	74%	
*meso*-tetrakis(1-methylpyridinium-4-yl)porphyrin	Sodium azide (100 mM)	T4 phage	90%	[107]
	L-histidine (100 mM)	T4 phage	78%	
5,10,15,20-Tetrakis(4-β- D-glucosylphenyl)porphyrin	1,3-diphenylisobenzofuran (0.1-5.0 mM)	T7 phage	42%	[64]
5,10,15-(4-β- D-glucosylphenyl)-20-phenylporphyrin	1,3-diphenylisobenzofuran (0.1-5.0 mM)	T7 phage	74%	[64]
Polyhydroxylated fullerene	β-carotene	T7 phage	69%	[57]
		PRD1 phage	56%	
	β-carotene (26 μM)	MS2 phage	50–60%	[90]
Rose bengal	Sodium azide (3.5–35 mM)	M13 phage	31%	[52]

##### 3.2.2.1. Singlet Oxygen in PDI of Mammalian Viruses

Singlet oxygen seems to be the most important mediator of virucidal activity (Table 3) on mammalian viruses. The rate of viral photoinactivation is significantly inhibited by oxygen removal or by addition of singlet oxygen quenchers, such as β-carotene, imidazole, L-histidine or sodium azide [45,84,106,107,108]. Hypericin may induce photochemical alterations on HIV major capsid protein p24, which are inhibited by sodium azide, suggesting that the damage results from singlet oxygen [111]. When merocyanine 540 [45], phthalocyanine derivatives [106] or rose bengal [108] were used as PS, the results suggest that ^1^O_2_ is the main cytotoxic species involved in VSV photoinactivation, while type I reactants such as hydroxyl radicals are less important.

##### 3.2.2.2. Singlet Oxygen in PDI of Bacteriophages

Considering the PDI of bacteriophages in the presence of singlet oxygen quenchers, the results (Table 3) suggest that, in most of the studied cases, singlet oxygen is an important mediator of the toxic effect induced by PDI. However, the participation of free radicals cannot be ruled out. For instance, the inactivation of M13 bacteriophage by MB was inhibited from 1.72 log to 0.54 log by sodium azide in a quencher dose-dependent mode, up to a concentration of 3.5 mM. However, photoinactivation occurred even in the presence of sodium azide, suggesting that both type I and type II mechanisms may be involved in the M13 photoinactivation process. In the presence of quencher concentrations ranging from 3.5 to 35 mM, a sodium azide protective effect was not observed, as evidenced by increasing rates of M13 phage photoinactivation, reaching a plateau thereafter [52]. Also, the effect of singlet oxygen quenchers and of hydrogen peroxide indicated singlet oxygen as the main factor responsible for the loss of biological activity of bacteriophage M13 by rose bengal [51].

The efficiency of 5,10,15-(4-β-D-galactosylphenyl)-20-(pentafluorophenyl)porphyrin to photoinactivate T7 phage decreased in 38% in the presence of sodium azide [87]. This result, and the ones obtained in the presence of DMTU (Table 2), proved that for this PS, both mechanisms play a role in T7 phage photoinactivation, with type I being the predominant one. Similar results were obtained by Gábor *et al.* [64] in the presence of glycoconjugated *meso*-tetraarylporphyrin derivatives as PS and using 1,3-diphenylisobenzofuran as the singlet oxygen quencher. When T7 phage was phototreated with 5,10,15,20-tetrakis(4-β-D-glucosylphenyl)porphyrin, the rate of inactivation decreased 42% in the presence of 1,3-diphenylisobenzofuran. When 5,10,15-(4-β-D-glucosylphenyl)-20-phenylporphyrin was used, the rate of protection substantially increased (74%). It can then be concluded that the type of PDI mechanism depends on the PS structure, with the symmetric derivative exerting its toxic effect mainly via the generation of free radicals, whether the asymmetric derivative proceeds mainly by singlet production [64]. However, in the study of Egyeki *et al.* [87] using the same asymmetric 5,10,15-(4-β-D-galactosylphenyl)-20-(pentafluorophenyl)porphyrin as PS, and the same phage, the toxic effect occurred mainly via free radical generation. Besides this, the contribution of type I and type II processes was PS concentration-dependent and the sum of the photoinactivation rate measured in the presence of scavengers was smaller than the one measured without the scavengers. This result may imply a synergism between singlet oxygen and hydroxyl radical-mediated damages or it can also be supposed that the efficiency of neither scavenger is 100% [64,87].

A recent study showed that irradiation of polyhydroxylated fullerene suspensions (40 μM) in the presence of β-carotene reduced the photoinactivation rate of PRD1 and T7 phages, demonstrating singlet oxygen involvement [57]. Also, when the T4-like phage was irradiated in the presence of porphyrin derivatives and singlet oxygen quenchers sodium azide and L-histidine, the rate of phage inactivation was considerably reduced, suggesting that singlet oxygen may be an important mediator of the virucidal activity of these PS [107]. However, from the data obtained, other inactivation mechanisms cannot be excluded [57,107].

Although some data about the importance of the type I and II mechanisms in PDI of bacteriophages are discrepant, in general, it seems that the type II pathway is more important than the type I mechanism in phage PDI. On the other hand, there are only a few studies focusing on the simultaneous effect of singlet oxygen and free radicals scavengers under the same protocol of viral PDI [64,84,87,90,106,107].

## 4. Molecular Targets of Antiviral PDI

The short-lived ROS generated by photodynamic mechanisms are responsible for the damage induced to critical molecular targets [112]. Different viral targets, such as the envelope lipids and proteins, capsid and core proteins and the nucleic acid can be attacked by singlet oxygen and/or other ROS (hydrogen peroxide, superoxide and hydroxyl radicals) to achieve the loss of infectivity [84]. For a better understanding of the photoinactivation process, the knowledge of how the molecular targets are affected by PDI assumes a great importance [113]. For this reason, a detailed photophysical and photochemical study of the interactions between the toxic species generated by the PS and key biomolecules such as lipids, proteins and nucleic acids is essential for the knowledge and prediction of photosensitization process efficiency [114]. However, the studies performed show that the primary target of PDI depends on the chemical structure of the PS, the targeted virus and the mechanism of photoinactivation [64]. 

### 4.1. Nucleic Acids

Depending upon the viruses, the nucleic acid can be either DNA or RNA (single or double stranded). The size of the nucleic acid also varies depending on the viruses. Several studies have shown that both DNA and RNA mammalian viruses and phages are efficiently inactivated by PDI. There is now considerable information that PS like MB can bind to and penetrate viral membranes, whereupon they intercalate with nucleic acids. Upon activation by light, the generated ROS can cause the destruction of the nucleic acids, particularly at guanine residues, preventing viral replication [115]. However, there is a difference in target selectivity depending on the mechanism involved: sugar moieties are usually attacked by radicals (generated via type I process) and guanine residues are the targets of singlet oxygen (generated via type II process) [97].

#### 4.1.1. DNA Damage

From the four DNA bases, guanine is the most susceptible component to suffer a type I photosensitization reaction, due to the fact that it exhibits the lowest oxidation potential among DNA bases and it is the only base that can be oxidized by singlet oxygen (type II process) [116].

The treatment of viruses with MB and other heterocyclic dyes resulted in the damage of viral DNA [53,65,75,76] either by base modification or base loss, single strand breaks (SSB), or cross-links of DNA with proteins [34,75,81,88,117]. It is known that cationic porphyrins can bind to nucleic acids via intercalation into base pairs or self-stacking, inducing lesions upon photoinactivation due to the easy oxidation of guanine residues [118,119,120].

The binding of cationic porphyrins to DNA is presumably due to the electrostatic interaction between the positively-charged substituents in the porphyrin macrocycle and the negatively charged phosphate oxygen atoms of DNA [120]. However, porphyrin binding to DNA is not a prerequisite for an efficient photosensitization, since free porphyrins are more effective in virus inactivation than the DNA-bound species [88]. This observation, which is in conflict with the generally accepted idea that the porphyrin molecule must be in close vicinity with the site of photosensitized damage, may be explained by the lower quantum yield of singlet oxygen by the bound porphyrin when compared with the free one [88].

##### 4.1.1.1. Damages in the DNA of Mammalian Viruses

Viral DNA is thought to be a critical target structure for PDI by MB and light [93]. DNA isolated from adenovirus treated with 1.3 μM MB exhibited a smear in Southern blot analysis, indicative of random DNA fragmentation [76]. MB plus light treatment of HSV-1 gives rise to DNA damage and blocks DNA replication [121]. 

##### 4.1.1.2. Damage in the DNA of Bacteriophages

An internal component of T4 phage has been suggested as an important target because MB needs to cross the outer barrier made by its protein capsids in order to produce a significant effect [65]. In fact, some of the irradiated phages could still inject functional genetic material but have lost their ability to form plaques, suggesting that their DNA was damaged. Protein synthesis was also severely impaired [65]. Treatment of M13 phage with MB and aluminum phthalocyanine tetrasulfonate (AlPcS_4_) caused strand breaks and piperidine-labile bonds in DNA, which is correlated with the loss of infectivity. This is in agreement with the proposal that lesions of the viral genome might be responsible for the lethality induced by sensitization [81]. DNA strand cleavage was found to be MB concentration and light dose dependent. Viral inactivation and DNA damage were found to be oxygen-dependent processes. However, DNA damage was not correlated with the loss of PM2 phage infectivity, as observed in transfection studies which measured the infectivity of the extracted viral DNA, indicating that DNA from MB-treated phage was just as capable of generating progeny virus as the untreated controls [53]. The observed DNA damage is not correlated with loss of phage infectivity and may not be the prime target of viral PDI, because 100% of closed circular DNA was recovered from the MB phototreated PM2 phage [53]. Concerning the effects of PDI on isolated viral DNA, treatment of M13mp2 DNA with increasing concentrations of MB, in the presence of light, yielded increasing amounts of 8-oxo-7,8-dihydro-2'-deoxyguanosine (8-oxodguo), a prevalent adduct produced by singlet oxygen and perhaps by oxygen free radicals. At 100 μM MB, 1 residue of 8-oxodguo was produced for every 40 residues of deoxyguanosine in DNA. Thus, treatment of M13mp2 DNA with MB plus light resulted in putative alterations at deoxyguanosine residues that impede the progression of DNA synthesis *in vitro* [116].

#### 4.1.2. RNA Damage

RNA has been suggested to be a key factor in viral PDI with many PS, but direct evidence of a correlation between RNA damage and infectivity loss has not been reported yet, as is the case of VSV when treated with phthalocyanine derivatives [81]. In RNA, as for DNA [71], guanine is suggested as the major target for oxidation by photosensitizing agents and light. 

##### 4.1.2.1. Damage in the RNA of Mammalian Viruses

VSV genome was damaged by 30 μg mL^−^^1^ of a chlorophyll derivative and red light illumination which caused a decrease of as much as 85% in RNA polymerase activity, which can be due to damage in the viral RNA polymerase complex, and 98% inhibition of viral RNA synthesis in 6 hours [77]. According to Moor *et al.* [82], the RNA and/or the RNA polymerase complex of VSV might be a major target for its photoinactivation by AlPcS_4_ and MB. MB and phthalocyanine derivatives inactivated VSV and inhibited fusion of the virus envelope with Vero cells. The degree of inhibition was small compared to the extent of virus inactivation, suggesting that non-membrane targets, like the viral RNA, might be involved in VSV photoinactivation. However, there is no report of a correlation between RNA damage and loss of infectivity [81]. Photoinactivation of HIV-1 by MB and light lead to destruction of its RNA [34].

##### 4.1.2.2. Damage in the RNA of Bacteriophages

Following MB plus light exposure, the Qβ RNA genome exhibited sufficient lethal lesions to account for phage inactivation [122]. However, the protein component of the phage also exerted some effect in viral PDI [122]. In a comparison of RNA photoinactivation using MB and rose bengal as the PS, Schneider *et al.* [54] suggested a causal relationship between 8-oxodguo formation in RNA and R17 and Qβ bacteriophage inactivation. However, no direct relationship between photodynamically induced RNA damage and viral inactivation was described [54]. 8-oxodguo formation or oxidative damage of Qβ RNA alone does not directly account for the lethal event of the virus. Directly treating extracted phage RNA with MB and light caused a loss of activity in the infectious RNA assay but there was a much greater loss of activity if the phage RNA was treated with MB and light in the phage *per se*. The results demonstrated that Qβ RNA infectious activity is significantly more affected by photoinactivation in its protein-associated virion state as compared with its purified isolated polymer state [92,122]. Inactivation of purified RNA by MB and light, in the absence of proteins, most likely occurs due to oxidative damage to the RNA at the site at which MB is bound and might involve oxidized bases such as 8-oxoguanine or strand breaks [122].

In spite of the reduced number of reports focusing on the damage induced by PDI in the nucleic acids of mammalian viruses and bacteriophages, it can be concluded that both DNA and RNA are potential targets of viral PDI. However, there are no studies specifically focusing on the damages induced to DNA and RNA of both mammalian viruses and bacteriophages under the same PDI protocol.

### 4.2. Outer Structures

Enveloped viruses are inactivated more rapidly than non-enveloped viruses because the destruction of the envelope structure is generally accompanied by loss of virus infectivity [13,40,94,123,124]. The damages caused by photodynamic reactions on unsaturated lipids present in their envelopes and/or on major envelope proteins, which act as PS binding-sites, modify their structure and avoid cell infection and virus replication [50,84]. However, some studies showed that non-enveloped viruses can also be efficiently inactivated by the toxic action of PS [55,56,58,62,64,65,66,67,73,81,87,88,94,122].

The higher susceptibility to PDI of enveloped viruses, relatively to non-enveloped viruses, indicates that the viral envelope may be a more important target than nucleic acids for photosensitization. It also indicates that the unsaturated lipids present in the envelope, as well as the major envelope proteins, are important PDI targets. However, as far as it is known, no studies focus on the degradation of viral envelope lipids after PDI or even on other viral internal lipids. There are, however, many studies about the effects of PDI on viral envelope proteins as well as on other core proteins.

The statement that enveloped viruses are more easily inactivated than non-enveloped ones is only based in indirect studies which compare the inactivation results of enveloped and non-enveloped viruses. The enveloped viruses used in PDI protocols [30,36,45,77,81,82,83] were only assayed for their protein alterations and no additional experimental work was done concerning their lipids. However, the results of PDI obtained by Lytle *et al.* [94] with the enveloped φ6 phage, although indirectly, are in good accordance with what is reported in the literature about the major contribution from lipids for the viral photoinactivation process. 

Relative to proteins degradation by PDI, the results of different studies showed that the main damage is the formation of protein cross-links, followed by other types of damage, which include loss of proteins, alterations in protein molecular conformation, mass and charge, and alterations in protein band intensity (Table 4).

When proteins are irradiated with UV or visible light in the presence of a PS, photooxidation of sensitive amino acid residues such as cysteine, L-histidine, tyrosine, methionine and tryptophan, and covalent cross-linking of peptide chains can be observed, leading to the formation of molecular aggregates [125,126], disrupting their normal folding conformation, thus forcing them into other conformations that affect their normal functioning [127]. In fact, the formation of cross-linked/aggregated material appears to be a major consequence of photosensitized-mediated protein oxidation [128], and it has been demonstrated that the formation of protein cross-links is not a primary photodynamic event, but a secondary reaction between the photooxidation products of sensitive amino acid residues and other groups in the protein [126].

The PS *per se* can induce alterations in the folding of some enzymes, leading to the exposure of some amino acid residues normally shielded in the protein, and to the shielding of others usually exposed in the molecule. These protein modifications lead to changes in properties such as solubility, proteolytic susceptibility, absorbance, and fluorescence emission of several of their amino acids. These alterations are mainly mediated by hydrogen peroxide and hydroxyl radical generation, although singlet oxygen mediated reactions could also occur [129]. The amino acids located in the surface of the protein are photooxidized at a much faster rate than the residues buried in the interior of the molecule. If a protein is completely unfolded, susceptible amino acids may also be attacked and photodegraded [103,130].

**Table 4:** Degradation of viral outer structures after mammalian viruses and bacteriophages PDI.

Virus	Type of damage	PS	Reference
**Enveloped-mammalian viruses**			
HSV-1	Viral envelope (reduced ability to adhere to and penetrate host cells)	Merocyanine 540	[45]
	Viral envelope (prevention of viral adsorption and host penetration)	Phthalocyanine derivatives	[131]
	Glycoprotein D; loss of proteins; dimerization; protein cross-links; alterations in protein molecular mass and charge	Phthalocyanine derivatives	[83]
HSV-2	Viral envelope (prevention of viral adsorption and host penetration)	Phthalocyanine derivatives	[131]
HSV	Protein cross-links	Phthalocyanine derivatives	[132]
VZV	Viral envelope (prevention of viral adsorption and host penetration)	Phthalocyanine derivatives	[131]
HIV	Major capsid protein p24	Hypericin	[111]
HIV-1	Loss of infectivity; loss of fusion function; membrane proteins cross-links	Hypericin	[30]
	Loss of infectivity; loss of fusion function; membrane proteins cross-links	Rose bengal	[30]
	p24 and gp120 proteins; protein cross-links	MB	[34]
	Inhibition of cell fusion activity of Env proteins	Natural and sulfonated tetraarylporphyrins	[36]
VSV	Loss of infectivity; loss of fusion function; cross-linking of G and M proteins	Hypericin	[30]
	Loss of infectivity; loss of fusion function; cross-linking of G and M proteins	Rose bengal	[30]
	Inhibition of fusion of the envelope to Vero cells; G protein	MB	[81]
	Inhibition of fusion of the envelope to Vero cells; G protein	Aluminum phthalocyanine tetrasulfonate	[81]
	G and M proteins; protein cross-links	Phthalocyanine derivatives	[82]
	G, M, L and N proteins; protein cross-links	Chlorophyll derivatives	[77]
Influenza virus	Loss of infectivity; loss of fusion function; cross-linking of G and M proteins	Hypericin	[30]
	Loss of infectivity; loss of fusion function; cross-linking of G and M proteins	Rose bengal	[30]
	Loss of infectivity; HA fusion protein; protein cross-links	Rose bengal	[108]
Sendai virus	Loss of infectivity; loss of fusion function; cross-linking of G and M proteins	Hypericin	[30]
	Loss of infectivity; loss of fusion function; cross-linking of G and M proteins	Rose bengal	[30]
Vaccinia virus	Histidine residues in virus proteins	Rose bengal	[86]
Human cytomegalovirus	Viral envelope (reduced ability to adhere to and penetrate host cells)	Merocyanine 540	[45]
Sindbis virus	Viral envelope (reduced ability to adhere to and penetrate host cells)	Merocyanine 540	[47]
	Viral capsid protein	Hypericin	[133]
Friend erythroleukemia virus	Viral envelope (reduced ability to adhere to and penetrate host cells)	Merocyanine 540	[134]
**Non-enveloped mammalian viruses**			
Adenovirus	Not damaged	Phthalocyanine derivatives	[131]
Enterovirus 71	Appearance/disappearance of protein bands; increase of the protein band intensity	Methylene blue	[85]
T7 phage	Protein capsid; loosening of the protein-DNA interaction	Glycoconjugated *meso*-tetraarylporphyrins	[64]
	Capsid and core proteins; loosening of protein-DNA interaction	Glycoconjugated *meso*-tetraarylporphyrins	[87]
	Capsid proteins; protein cross-links	*meso*-Tetrakis(1-methylpyridinium-4-yl)porphyrin	[88]
	Capsid proteins; protein cross-links	Polyhydroxylated fullerene	[57]
M13 phage	Coat protein	Methylene blueAluminum phthalocyanine tetrasulfunate	[81]
PRD1 phage	Capsid proteins; protein cross-links; phospholipids (less affected)	Polyhydroxylated fullerene	[57]
Qβ phage	Coat and maturation (A) proteins; formation of protein carbonyls; RNA-protein cross-links	Methylene blue	[89]
	RNA-protein cross-links	Methylene blue	[92]
MS2 phage	A protein	Polyhydroxylated fullerene	[57]

#### 4.2.1. Damage on Mammalian Viral Outer Structures

It has been shown that enveloped viruses can be inactivated due to protein damage [30,82,83,131]. However, while the same treatment is reported to be ineffective against some non-enveloped viruses [83,131], the results from Wong *et al.* [85] showed that even a non-enveloped virus can be efficiently inactivated due to the damage induced by PDI to its viral proteins (Table 4).

The proteins in the viral envelope of HSV-1 were considered to be major targets of merocyanine 540 photosensitization [45]. Some phthalocyanine derivatives have been shown to induce cross-links in HSV protein that might be responsible for the observed loss of infectivity [132]. Protein analysis by SDS-PAGE, after treatment with phthalocyanine derivatives, revealed irreversible changes in the HSV-1 envelope proteins, which were reflected by the loss of many proteins, the appearance of cross‑linked material on the top of the gel and by alterations in the molecular mass and molecular charge of the proteins. These alterations contribute, in all likelihood to HSV-1 inactivation [83]. 

In VSV treated with 3.75–30 μL mL^−1^ of chlorophyll derivatives and light, the M protein band was not detected, which was accompanied by a decrease in the intensity of the G protein band [77]. Large complexes of proteins were also detected on the top of the gel, indicating that viral PDI cross-linked the proteins [77]. Using a fusion assay and protein analysis, it was shown that MB and AlPcS_4_ caused a decrease in the intensity of the G-protein (which is known to play a crucial role in binding VSV to the host cell) band and a slight decrease in the intensity of M protein (matrix protein) band and protein cross-links. However, the observed damage in viral proteins could not account for VSV PDI [82]. VSV was inactivated by MB and phthalocyanine derivatives, which inhibited the fusion of the virus envelope to Vero cells. However, the degree of this inhibition was small compared to the extent of virus inactivation (43% inhibition *vs.* 4.7 log or 99.998% inactivation, for MB) [81]. Abe and Wagner [81] also found few changes in the relative abundance of VSV G protein after MB and AlPcS4 phototreatment, and they also observed additional protein bands on SDS-PAGE analysis [81]. It was found, by Western blot analysis, that HIV-1 p24 and gp120 proteins were altered in size, possibly due to cross-linking, after MB phototreatment [34]. However, using the same PS, AlpcS_4_ and MB, no changes in protein patterns after SDS-PAGE of the viral proteins were observed, under conditions that caused complete VSV inactivation [135].

The results from Vzorov *et al.* [36] indicated that the porphyrins inhibited the cell fusion activity of HIV Env proteins (a biological function that is important for viral entry as well as induction of viral cytopathic effects) when expressed from recombinant vectors. These results showed that the viral Env protein is an important target of these compounds [36].

PDI of influenza virus by rose bengal altered the HA fusion protein and led to protein cross-links [108].

Photoinactivation of vaccinia virus with rose bengal significantly altered the concentration and oxidized histidine in vaccinia virus protein, suggesting that inactivation was attributed to alterations in viral proteins, as opposed to nucleic acids [86].

Treatment of of influenza and Sindbis viruses by hypericin [30], lead to an extensive cross-linking of the envelope proteins, which may have impaired the capacity of the viruses to adhere to and penetrate the host cells.

The protein profile of the non-enveloped enterovirus 71 was considerably altered after a low dose PDI and a MB concentration ≥0.5 μM, as revealed by a smearing and the disappearance of several protein bands [85]. However, enterovirus 71 PDI was also due to damages in the viral genome [85]. 

#### 4.2.2. Damages on Bacteriophage Outer Structures

In spite of the limited available data for enveloped bacteriophages, substantially higher photoinactivation rates compared with other non-enveloped phages were described [94]. The photoinactivation by merocyanine 540 of four bacteriophages, two non-enveloped phages without lipids (phi X174 and T7), a non-enveloped phage with lipids (PRD1), and an enveloped phage with an external lipoprotein envelope (phi 6) was studied by Lytle *et al.* [94]. The survival curves of the different viruses clearly demonstrated different levels of sensitivity to photoinactivation by this PS, with phi 6 being the most sensitive, followed by T7 (21-fold less sensitive). While both PRD1 and phi 6 have lipid components, only phi 6 was photoinactivated by the PS. Thus, the internal lipid components of PRD1 were not sufficient to allow photoinactivation by merocyanine 540. A higher inactivation rate with a fullerene derivative was also observed by Hotze *et al.* [57] for a phage without lipids (T7 phage) than for PRD1 phage. The dissimilarities in phage composition resulted from differential resistance to singlet oxygen by the outer structures, since PRD1 has a double capsid with an internal lipid membrane, whereas T7 has a single proteinaceous capsid lacking lipids, and both phages contain double stranded DNA with similar GC content (48% for T7 and 51% for PRD1) [57]. Phage proteins were significantly affected by photosensitization (30–92%) when compared to the relatively smaller effect on nucleic acids in both PRD1 and T7, and lipids in PRD1 phage (≤13%), as assessed by FTIR spectra analysis [57]. The higher T7 phage inactivation is consistent with greater damage to its proteinaceous capsid. Besides this, SDS-PAGE analysis further evidenced that oxidative cross-linking of capsid proteins induced by exogenous singlet oxygen is the likely cause of phage inactivation [57]. The high propensity for MS2 phage inactivation by this PS (compared to PRD1 and T7 phages) possibly arises from damage to its A protein, which is necessary for infecting its host *Escherichia coli* since it contains highly reactive amino acids such as methionine, cysteine, histidine, and tyrosine and not to damages to the nucleic acid [57]. Glycosylated substituted porphyrins led to structural changes at the protein capsid and/or loosening of the protein-DNA interaction, which can be responsible for T7 phage inactivation [64]. Besides of the alteration of the DNA structure, the phototreatment pointed to significant alterations in the protein structure and/or in the DNA-protein interaction, which may be the cause of photodynamic inactivation [87,88]. The alterations in the DNA secondary structure might also be the result of photochemical damage in phage capsid proteins and consequent disruption of the phage particle. Photomodification of core proteins can also lead to phage inactivation, even if the primary structure of the DNA part is preserved, since these proteins play an important role in the early events of infection and DNA penetration [87]. The damage of T7 nucleoprotein is a complex process and clearly both phage DNA and protein capsid are affected by photoreactions [88]. Irradiation of Qβ bacteriophage in the presence of increasing concentrations of MB resulted in exponentially increasing amounts of viral RNA-protein cross-linkage products, and this is probably the most important event in viral inactivation [92]. The RNA genome of Qβ bacteriophage contained sufficiently lethal lesions following MB plus light exposure to account for the resulting phage inactivation. Nevertheless, the data also indicate that the protein component of the phage somehow contributes to the inactivation of the phage [122]. The protein component of Qβ phage is involved in the process of photoinactivation because the formation of protein carbonyls and RNA-protein cross-links were efficiently formed by MB plus light exposure [89]. The close correlation of cross-link formation with phage inactivation and the expectation that even one such cross-link in a phage genome would be lethal makes the RNA-protein cross-link lesion a strong candidate for the primary inactivating lesion of Qβ phage exposed to MB and light [122]. 

Little alteration of M13 phage proteins on SDS-PAGE after MB and AlPcS_4_ photoinactivation was observed by Abe and Wagner [81]. The results of Zupán *et al.* [136], suggested that the tetracationic porphyrin *meso*-tetrakis(1-methylpyridinium-4-yl)porphyrin did not interact with capsid proteins and did not disturb protein-DNA interaction, even if it has a strong stabilization effect on the intraphage DNA.

## 5. Resistance to PDI and Recovery of Viability

The development of increasing numbers of antiviral agents over the past decades, in the same way as with antibiotics, has provided the clinician with therapeutic options previously unavailable. With the increasing utilization of antiviral drugs, however, has come an enhanced appreciation of the development of antiviral resistance [1,7,137,138,139,140]. Drug resistance is costly to the health service, to the patient who fails to gain maximum therapeutic benefit, and for the community in which resistant viruses may be spread [9]. 

There is now an urgent need for the development of novel, convenient and inexpensive measures for combating antimicrobial-untreatable infections and limiting the development of additional antimicrobial resistant microorganisms. Photodynamic technology may provide one approach to meet this need, both in terms of therapy and in terms of sterilization, by a mechanism that is markedly different from that typical of most antimicrobials [1,141,142].

As mentioned before, photosensitization involves the generation of singlet oxygen and free radical species, which cause molecular damage. Whether microorganisms could develop resistance to these active oxygen species is still questionable [143] and, consequently, the development of microbial resistance to photosensitization is still under debate. Until now, the development of microbial resistance to PDI is not known and is thought very improbable to be developed. In general, the development of resistance to PDI by microbial strains should be considered as an unlikely event since this process is typically multi-target, with ROS causing damage to many microbial components, which is at a variance with the mechanism of action of most antimicrobial drugs [139,144,145]. In contrast to most common antimicrobials, the number of molecular alterations required to ensure survival would be too great and the microorganism would require multi-site mutations to become highly resistant, an event with significantly lower probability than single-site mutations, which is often sufficient for conferring resistance to small-molecule inhibitors [42,146]. This particular property of antimicrobial PDI is important regarding the repeated treatment of chronic and/or recurrent infections [139].

Antimicrobial PDI, when compared to standard treatments which may require application for several weeks to achieve an effective killing of the microorganism, shortly after initiation of light exposure, exhibits serious and irreversible damage of microorganisms [66,68]. This damage does not allow the creation or operation of any kind of anti-drug or mutagenic mechanism. Antimicrobial PDI is therefore very effective and, up until now, no photosensitization-resistant mutants have been found [68].

### 5.1. Resistance of Mammalian Viruses and Recovery of Viability after Photosensitization

Data from North *et al.* [33] show that HIV azidothymidine (AZT)-resistant strains were as susceptible as the AZT-sensitive ones to photosensitization with a benzoporphyrin derivative. This finding comes as no surprise since the mechanisms of action of AZT (inhibition of reverse transcription) and light-activated benzoporphyrin derivative are different. Thus, mutations in the virus that occur at the reverse transcriptase level will not affect photodynamic destruction [33].

Studies focusing on the possible development of viral resistance are extremely scarce and little is known about the recovery of viral viability after consecutive photodynamic treatments.

### 5.2. Bacteriophage Resistance and Viability Recovery after Photosensitization

Concerning bacteriophages, there is only one study focusing on the possible development of viral resistance after photosensitization [68]. After 10 consecutive cycles of photodynamic treatment, a T4-like phage, in the presence of the tricationic porphyrin 5-(pentafluorophenyl)-10,15,20-tris(1-methylpyridinium-4-yl)porphyrin (Tri-Py^+^-Me-PF) at 5.0 μM under white light irradiation, exhibited no changes in the rate of photoinactivation during the course of the experiments, meaning that no resistance was observed. If phage resistance would occur, important reductions on phage photoinactivation efficiency would be detected between experiments. Besides that, T4-like phage did not recover its viability after exposure to Tri-Py^+^-Me-PF during 120 min of irradiation [68]. In a preliminary study by Perdrau and Todd [12], all attempts at reactivating the inactivated *Staphylococcus* phage by MB were unsuccessful. 

## 6. Factors Affecting Viral PDI

### 6.1. Effect of the Number of Charges, Symmetry, Size of Meso Substituent Groups and Photosensitizer Concentration

It has been shown that the location and binding site of the PS, which is highly dependent on the structure and intramolecular charge distribution, is an important factor in microbial PDI [143,147].

In terms of molecular structure, molecular charge is important in determining antimicrobial activity. Positively charged PS are generally more efficient and can act at lower concentrations than neutral and anionic PS molecules [144]. The positive charges on the PS molecule appear to promote a tight electrostatic interaction between the positively charged PS and the negatively charged sites at the viral capsids and envelopes, orientating the PS toward sites which are critical for the stability and metabolism of a particular microorganism [44,147,148]. This kind of association increases the efficiency of the photoinactivation process.

Cationic PS photodamage can be induced in nucleic acid or viral outer structures by PS binding or by PS localized in its vicinity [136]. For instance, it is more likely that positively charged PS will be effective in causing nucleic acid damage than will neutral or anionic congeners, which mainly act against the outer side of the microorganism [149].

The symmetry and the size of the chain of *meso* substituent groups also affect the photodynamic effect. PS with opposite charged groups are more symmetrical than PS with adjacent charged groups. The adjacent positive charges in the PS macrocycle should result in a molecular distortion due to electrostatic repulsion [150]. The toxicity of a PS can be modulated by the introduction of selected substituents on the macrocycle periphery. In this way, the physicochemical properties of a synthetic PS can be manipulated in order to enhance its interactions with the structural features of the viruses, such as viral capsids, and to minimize the interactions with plasma membranes or mammalian cell membranes [44]. 

The amphiphilic nature of a PS is another important feature affecting PDI efficiency and can be modulated by the introduction of adequate functionalities in the macrocycle periphery, such as different numbers of positive charges, an asymmetrical charge distribution, or introduction of aromatic hydrocarbon side chains [16,151]. 

PS concentration is also an important parameter that must be taken into account since viral PDI was shown to be strongly influenced by PS concentration. Increasing the PS concentration reduces the time needed to achieve complete viral inactivation, thus increasing the efficiency of a particular PDI protocol [66].

#### 6.1.1. Mammalian Viruses PDI

Complete inactivation of VSV (4.2 log) can be obtained by treating it with 1.0 μM of the anionic phthalocyanine derivative AlPcS_4_ and 5 min illumination with red light. For the neutral phthalocyanine derivative (Pc_4_), complete inactivation (4 log) was achieved using a much lower amount of PS (4.5 nM) in combination with 10 min illumination [82]. The inactivation of VSV in PBS showed a linear relationship with illumination time [82]. Inactivation of the fusion activity of VSV, influenza and Sendai viruses was reached with nanomolar concentrations of hypericin and rose bengal and was absolutely dependent upon light and increased with increasing time of illumination [30]. HAV in PBS or plasma was completely inactivated within 10 min (>3.7 log) by the cationic symmetric porphyrin *meso*-tetrakis(1-methylpyridinium-4-yl)porphyrin. In contrast, inactivation of HAV to 3.6 log with the anionic symmetric porphyrin *meso*-tetrakis(4-sulfonatephenyl)porphyrin required 90 min [44]. The rate and extent of inactivation appeared to vary with the nature of the *meso* substituent groups [44]. HIV and VSV lost infectivity upon illumination with hypericin and rose bengal in a concentration-dependent manner [30].

#### 6.1.2. Bacteriophage PDI

MS2 phage inactivation has been observed with neutral porphyrin derivatives. However, this required higher irradiation periods (30 min) than for the cationic ones (1 min) [44]. Neutral glycosylated substituted porphyrins can also significantly photoinactivate the T7 phage [64,87]. The T4-like phage PDI was achieved by exposing the phage in the presence of six cationic porphyrins at different concentrations (0.5, 1.0 and 5.0 μM) to white light for 270 min. The results showed that phage photoinactivation varied according with the PS concentration, with higher concentrations being the most efficient ones [66]. The T4-like phage PDI also varied with the number of porphyrin charges, with tri- and tetracationic porphyrin derivatives being more effective in viral inactivation that the dicationic ones, which inactivated the phage below the limit of detection. Tetra- and tricationic porphyrin derivatives (*meso*-tetrakis(1-methylpyridinium-4-yl)porphyrin and 5-(pentafluorophenyl)-10,15,20-tris(1-methylpyridinium-4-yl)porphyrin, respectively) lead to complete T4-like phage inactivation (~7 log) after 270 min of irradiation with 40 W m^−2^ [66]. This tetracationic porphyrin showed similar results in another study (7 log of reduction) for lambda phage inactivation, when irradiated with light of 658 nm [58]. Increasing porphyrin concentration at a fixed light dose leads to increased viral inactivation [58]. A concentration-dependent effect was also detected with a porphyrin derivative [87], but over 2.0 μM of PS the process was saturated. A further increase in porphyrin concentration did not lead to a higher inactivation rate of T7 phage. Aggregation and/or photobleaching of PS are likely explanations [87]. Cationic *meso*-tetrakis(1-alkylpyridinium-4-yl)porphyrin derivatives with different alkyl substituent groups were tested for MS2 phage inactivation but, with the exception of 5,10,15,20-tetrakis(4-sulfonatophenyl)porphyrin, showed toxicity even in the absence of light [44]. 

In a study conducted by Gábor *et al.* [64], the porphyrin derivative with symmetrical glycosylated groups was found to be twice as effective as the asymmetrical one on the inactivation process of T7 phage. According to Costa and colleagues [66], the rate of T4-like phage inactivation was also dependent on the lipophilic character of the *meso*-substituent groups. The presence of a lipophilic aryl group in one of the *meso* positions of the porphyrin core appears to have an important role in phage inactivation, affecting the rate and efficiency of T4-like phage [66]. Casteel *et al.* [44] have also observed differences in the photoinactivation rate of MS2 phage when they used PS with different alkyl substituent groups and concluded that the rate and extent of inactivation appeared to vary with the nature of the *meso* substituent groups.

### 6.2. Effect of Different Light Sources and Fluence Rate on Antimicrobial PDT

PDT requires a source of light to activate the PS by exposing it to visible or near-visible light at a specific wavelength [152]. The light source for PDT must also exhibit suitable spectral characteristics coinciding preferentially with the maximum absorption wavelength range of the PS, applied in order to generate enough ROS to produce an efficient toxic effect [153].

In parallel with the advances in chemistry (related with the discovery and synthesis of new and more efficient PS) there has also been much activity in developing new light sources, better suited for the photosensitization process. Briefly, these include user-friendly lasers frequently based on solid state laser diodes, as well as inexpensive light emitting diodes (LED) and filtered broad-band lamps [154]. 

PS activation has been achieved via a variety of light sources, such as arc plasma discharge lamps, metal halogen lamps, slide projector illumination assemblies, and a variety of lasers. For treatment of larger areas, non-coherent light sources, such as tungsten filament, quartz halogen, xenon arc, metal halide, and phosphor-coated sodium lamps, are in use. Recently, non-laser light sources, such as LED, have also been applied in PDT. These light sources are much less expensive and small, lightweight and highly flexible, its lifetime can reach up to one hundred thousands hours, and can be manufactured to wavelengths that activate commercially available PS [152,155,156,157,158,159].

At first glance, the available literature on fluence rate effects for PDT seems contradictory. Some studies indicate less damage at low fluence rate, others indicate more killing at lower, compared to higher, fluence rates for the same total fluence and some indicate no influence of fluence rate at all [152,157,158]. A reduction in the fluence rate lowers the rate of oxygen consumption, thereby extending the radius over which singlet oxygen may be formed and consequently increasing the phototoxic effect [159]. Qin *et al.* [160] showed that an increase in the fluence rate increases microbial damage, although, it seems to have an upper limit of photons to observe this effect. Since each PS molecule can only absorb one photon at a time, when the number of light photons bypasses the number of PS molecules, the PS will no longer be able to absorb the photons “in excess” and the rate of PDI will not increase. In fact, if the number of photons is higher than this limit, the antimicrobial effect will decrease because the dye in suspension will not absorb all the excess light [160]. Schindl *et al.* [161] referred that the biological effect of light depends on the fluence, irrespective of the time over which this dose is delivered. Maclean *et al.* [162] also indicate that the inactivating light may be applied at high irradiance over a short time or at lower irradiance over a longer time. A numerical model, assuming that the rate of photodynamic damage occurring at time *t* is proportional to the fluence rate at that time and the local concentrations of PS and oxygen can be established. However, according to this model, relatively low fluence rates can be nearly as effective as high fluence rate sources if applied over the same period of time [163].

There is also a direct correlation between the phototoxic effect and the PS concentration and light fluence. With a lowering of the PS concentration, more light has to be applied to achieve identical effects, and *vice versa*. Lower doses of PS require higher activating light fluences, and higher fluence requires a longer duration of light application [96]. 

#### 6.2.1. Effect of Light on Mammalian Viruses PDI

The effects of dengue virus inactivation were increased with the increase of MB concentration, the enhancement of power density of the light source and the extension of illumination time, as well as the decrease of illumination distance. This enabled the narrow bandwidth light system to kill or inactivate the enveloped virus at much greater distance in much shorter time [74]. VSV in the presence of MB was rapidly inactivated by red (provided by LED incident light at 272 W cm^−^^2^) or green-yellow light (provided by low-pressure sodium lamps at a fluence rate of 165 W cm^−^^2^) but slower by white light (provided by a bank of fluorescent tubes at a fluence rate of 42 W cm^−^^2^) [46], showing that higher power densities produce a high rate of viral inactivation than low fluence rates. Wagner *et al.* [164] also showed that red light of 9 W m^−^^2^, given at a total dose of 1.8 × 10^4^ and 3.2 × 10^4^ J m^-2^, inactivated MB-treated VSV by 6 and ≥7 log, respectively. VSV inactivation was linearly dependent on the fluence rate of red light illumination [165]. 

#### 6.2.2. Effect of Light on Bacteriophage PDI

In terms of what is known about phage PDI, only one study focusing on the effect of different light sources and power densities [67] exists. In this study, cationic porphyrin derivatives (*meso*-tetrakis(1-methylpyridinium-4-yl)porphyrin and 5-(pentafluorophenyl)-10,15,20-tris(1-methylpyridinium-4-yl)porphyrin), when irradiated with different sources of light (fluorescent PAR lamps, sun light and halogen lamp) with fluence rates ranging from 40 W m^−2^ to 1690 W m^−2^, efficiently photoinactivated non-enveloped phages. All light sources tested lead to reductions of about 7 log for the somatic T4-like phage. However, the rate and the extent of inactivation were dependent on the light source, namely when low fluence rates were used (40 W m^−2^) and on the energy dose, being considerably more effective when light was delivered at a lower fluence rate. However, depending on the light source used, different irradiation periods were required to inactivate T4-like phage to the limits of detection. The results also showed that the efficacy of T4-like phage inactivation, using the same fluence rate, was dependent on the light source used, in particular when the light is delivered at a low fluence rate. M13 phage was phototreated with 5.0 μM MB and was inactivated in an irradiation dose-dependent manner [52]. Kastury and Platz [58] showed that increasing the concentration of a PS at a fixed light dose leads to increased viral inactivation as does an increase in the total light exposure at a fixed PS concentration. The inactivation rate of T1 bacteriophage increased with increasing fluence rate, indicating that the distance of the sample from the light source is a variable which must be controlled [73]. At higher PS concentrations, the inactivation rate reaches a maximum and then decreases, because the filtering effect of the dye decreases the effective fluence rate [73]. In a simple model purposed by Lee *et al.* [56], the phage survival ratio can also be considered as a decreasing exponential fraction of the light fluence (assuming that the fluence is uniform throughout the system).

## 7. Conclusion

The efficiency of different types of PS in viral PDI has been proved for different types of mammalian viruses and bacteriophages, whether they are enveloped or non-enveloped, for either DNA or RNA viruses. Even though enveloped viruses are more easily inactivated than non-enveloped ones, several studies confirm that non-enveloped mammalian viruses and phages can be efficiently inactivated by PDI. The type of viral nucleic acid has not been described as an important factor affecting viral photoinactivation but, as far as it is known, no studies specifically focus on the photoinactivation behaviour of DNA and RNA viruses. However, RNA phage MS2 was highly susceptible to photoinactivation when compared with DNA phages under the same conditions of photosensitization.

The type of mechanisms involved in the process of viral photosensitization was already elucidated and singlet oxygen and free radical species were identified as important contributors for an effective viral PDI. However, the contribution of singlet oxygen seems to be more pronounced in mammalian viruses and bacteriophage PDI. There are, however, few studies simultaneously comparing the contribution of both types of mechanisms (type I and type II) involved in viral PDI. The primary targets for the photoinactivation of viruses, whether treating mammalian viruses or phages, are the outer structures. Although there are several studies about the specific effects of PDI on viral proteins, for different types of mammalian viruses and phages, there are no studies concerning the specific effects of PDI on viral lipids. However, it has been clearly shown that enveloped viruses are more easily inactivated than their non-enveloped counterparts, which imply that the lipids present on viral envelopes are important targets of viral PDI.

PS are effective in inactivating the phages to the limits of detection in a way that they do not recover viability, avoiding the development of viral resistance. Nothing is known yet for the particular case of mammalian viruses but, as the viral targets are the same for mammalian viruses and phages, it is also expected that no resistance will be developed in the case of mammalian viruses. Besides that, antiviral PDI is equally effective whether the mammalian virus is sensitive or resistant to conventional antiviral agents. Taking into account all these advantages, PDI for viral inactivation can be regarded as a promising alternative therapy to conventional antiviral treatments, namely for the disinfection of blood and blood products, preventing viral contamination and for the treatment of wound and burn infections. Viral PDI has a fast mode of action and has also the additional benefits of being more economical and an environmental friendly technology, which might be successfully used also in the environmental field for wastewater, drinking water and fish-farming water disinfection.

Different PS concentrations and different light sources and fluence rates were tested, showing that they are important PDI parameters that must inevitably be taken into account when a viral photosensitization protocol has to be elaborated. The inactivation of mammalian viruses and phages can be attained at micromolar-level PS concentrations and different light sources are equally effective, depending on the final dose at which the viruses are exposed to. Besides that, PS can also be modulated by the addition of different *meso* substituent groups and positive charges in order to facilitate their interactions with the viruses, making them more efficient for mammalian viruses and phage PDI.

The similarity of the results obtained for mammalian viruses and bacteriophages show that they exhibit a similar behaviour when submitted to viral photoinactivation techniques: (i) the PS used for viral PDI were equally effective in the photoinactivation of mammalian viruses and bacteriophages; (ii) the mechanism of mammalian viruses and bacteriophage photosensitization involves the production of singlet oxygen (type II mechanism) with a slight contribution of free radical species (type I mechanism); (iii) singlet oxygen and free radicals were shown to affect viral nucleic acids and also the proteins and lipids present in the mammalian viruses and bacteriophage outer surfaces, with the latter being considerably more affected by PDI; and (iv) the rate and extent of mammalian viruses and phage PDI is also affected by the same factors, like the PS concentration and number of positive charges, the nature and position of *meso* substituent groups, the fluence rate and energy dose. Consequently, it is important to persist in the development of more PDI phage studies to clarify some aspects of viral PDI, such as influence of viral nucleic acid type (DNA or RNA) in the photoinactivation efficiency and the possibility of viral resistance development and viability recovery after photosensitization. It will also be important to study the synergistic effect between viral PDI and antiviral classical methodologies using bacteriophages as models of mammalian virus’ photoinactivation.
